# Toxocariasis as an Elderly Zoonosis: Seroprevalence, Neurocognitive Assessment, and Associated Risk Factors in Persons 50 Years and Older

**DOI:** 10.3390/pathogens14111095

**Published:** 2025-10-28

**Authors:** Gabriela Geraldi da Silva Rapchan, Isabella Braghin Ferreira, Viviane dos Santos Vaccaro Lima, Susana Angélica Zevallos Lescano, Giovanna Ribelatto Monteiro, Gustavo Cardoso dos Santos, Larissa Sapucaia Ferreira Esteves, Fabiano Borges Figueiredo, Louise Bach Kmetiuk, Alexander Welker Biondo, Rogerio Giuffrida, Vamilton Alvares Santarem

**Affiliations:** 1 Graduate College in Animal Sciences and Health Sciences, University of Western São Paulo (UNOESTE), Presidente Prudente 19067-175, SP, Brazil; gabriela_geraldi@yahoo.com.br (G.G.d.S.R.); braghinisabella@hotmail.com (I.B.F.); viviane.vaccaro@hotmail.com (V.d.S.V.L.); gimonteiroo1501@gmail.com (G.R.M.); gucardoszo@gmail.com (G.C.d.S.); larissaesteves@unoeste.br (L.S.F.E.); rgiuffrida@unoeste.br (R.G.); vamilton@unoeste.br (V.A.S.); 2 Institute of Tropical Medicine of São Paulo, University of São Paulo (USP), São Paulo 05403-000, SP, Brazil; suzeles@usp.br; 3 Laboratory of Cell Biology, Carlos Chagas Institute, Curitiba 81310-020, PR, Brazil; fabiano.figueiredo@fiocruz.br (F.B.F.); louisebachk@gmail.com (L.B.K.); 4 Department of Veterinary Medicine, Federal University of Paraná (UFPR), Curitiba 80035-050, PR, Brazil

**Keywords:** epidemiology, infection, old age, *Toxocara* spp., zoonosis

## Abstract

Toxocariasis, a geohelminthiasis caused by *Toxocara canis* and *Toxocara cati* nematodes, has an estimated 19% seroprevalence worldwide. Although children have been considered more prone to infection, adults and the elderly may also be considered at risk. Accordingly, the present study aimed to assess the associated risk factors for *Toxocara* spp. seropositivity in a population of 290 individuals over 50 years old, attended by the Public Health System in western São Paulo state. Socioepidemiological information was obtained by a semi-structured questionnaire, a blood (serum) sample, and a neurocognitive function assessment by the 10-point cognitive screener (10-CS). Overall, 89/290 (30.7%; 95% CI: 25.7–36.2%) individuals presented anti-*Toxocara* spp. IgG antibodies by ELISA. Multivariate analysis revealed that individuals raising both dogs and cats were 3-fold more likely to be seropositive than the ones without any pet (*p* = 0.002), while educational level resulted in an associated protective factor (*p* = 0.001). No seropositivity influence was observed for the other evaluated variables, including age, gender, monthly income, consumption of drinkable water or raw/undercooked meat, having a dirty floor at home, contact with soil, washing vegetables and hands before meals, and onychophagy. Although seropositivity was not statistically associated with dementia (*p* = 0.198) and neuropsychiatric disorder (*p* = 0.440), results herein indicated toxocariasis as a likely under-reported and neglected infection in the older human population. As an immunosenescence risk group that should be continuously monitored, elderly pet owners may be at risk and should be extra careful with self-hygiene and pet deworming, particularly when owning multiple pet species, to reduce the risk of toxocariasis infection.

## 1. Introduction

Human toxocariasis has been mainly transmitted by contaminated soil ingestion with eggs of *Toxocara canis* and *Toxocara cati*, shed into the feces of definitive hosts, respectively, dogs and cats [1]. As a zoonotic helminthic infection, toxocariasis has been classified amongst the most neglected parasitic diseases demanding public health action, along with toxoplasmosis, Chagas, cysticercosis, trichomoniasis, and cyclosporiasis, according to the Centers for Disease Control and Prevention [2]. The disease has been described to primarily impact lower socioeconomic populations living in countries with tropical and subtropical climates [3].

Although infections have been mostly asymptomatic and subclinical, migrating *Toxocara* spp. larvae may lead to non-specific symptoms or cause visceral, ocular, or neurological (neurotoxocariasis) toxocariasis, depending on the affected organ [4]. Parasitic agents, such as *Toxocara* spp., can be linked to cognitive decline and changes in human behavior [5], and is statistically associated with the worsening of working memory, sustained attention, and processing speed [6]. Toxocariasis has been associated with cognitive impairment and dementia in women [7] and men [8] from Germany, and in women from Taiwan [9]. In addition, seropositivity for anti-*Toxocara* IgG was significantly associated (OR 1.93) with Alzheimer’s disease (AD) in an Iranian case–control study [10]. Nevertheless, AD-like pathology triggered by infection with *T. canis* L3 larvae was experimentally refuted in an observational murine study over a 16-week post-infection timeline [11].

According to the Alzheimer’s Disease International Organization (ADI), over 55 million people may be presenting with dementia worldwide, with around 60% living in low- or middle-income countries, which may be doubling every 20 years, reaching 78 million unhealthy people in 2030 [12]. In Brazil, around 2 million people have been affected by some kind of dementia, mostly (77%) undiagnosed [13]. Nonetheless, underestimation should be considered due to misdiagnosis associated with limited financial resources and a lack of experts [14]. The low average educational level in Brazil may also impair the proper diagnosis, as screening tests for dementia and cognitive disorders have been adapted from high-income countries [15].

Dementia, or major neurocognitive disorder, has been characterized by a significant decline and progressive loss in at least two cognitive domains (executive function, complex attention, language, learning, memory, perceptual–motor, or social cognition) and may affect the social and occupational function of a given person [16]. In addition, such decline may characterize a change from a patient’s prior level of cognitive ability, which may persist and progress over time [17]. The most common cause of dementia has been Alzheimer’s multifactorial disorder [12], which has been associated with several risk factors including aging, genetic predisposition, traumatic brain injury, environmental variables, and vascular and infectious diseases [18]. 

Although neurotoxocariasis has been well described in adults, seropositivity and the associated risk factors of toxocariasis, particularly regarding cognitive function, in persons 50 years or over remained to be fully established. Accordingly, the present study aimed to assess the associated risk factors for the seropositivity of anti-*Toxocara* spp. antibodies, including the association between these antibodies and neurocognitive function, in a population aged 50 years or older and attended by the Public Health System in a major city of southeastern Brazil.

## 2. Materials and Methods

### 2.1. Ethics Statement

The study was approved by the National Commission of Ethics in Research (CAAE: protocol 75971523.5.0000.5515), University of Western São Paulo (UNOESTE), Presidente Prudente, São Paulo, Brazil. 

### 2.2. Study Design, Population, and Sampling

**Figure 1:**
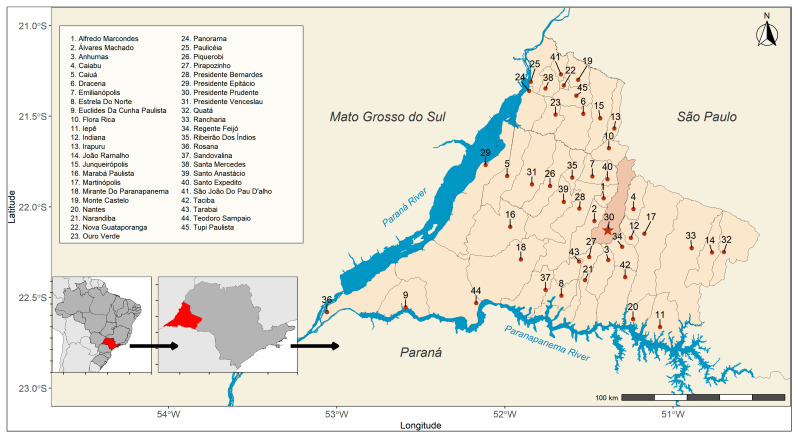
Municipalities (*n* = 45) that make up the Regional Health Care Network 11 (RRAS 11) at the triple border of the state of São Paulo with Paraná and Mato Grosso do Sul, Brazil. The points represent the municipal seats. The highlighted municipality serves as the regional reference (Presidente Prudente).

The study herein was a cross-sectional serosurvey of anti-*Toxocara* spp. antibodies (IgG) and the associated risk factors conducted from February to December 2024 in 290 individuals aged 50 years and older who underwent routine medical examinations at the public Brazilian Unified National Health System in the Presidente Prudente city, western São Paulo state, southeastern Brazil (Figure 1). In 2024, the population of the studied area was estimated to be 765,208 individuals, including 255,675 aged over 50 years (33.4%). Regarding the targeted population, 54.2% were estimated as women and 45.8% men [19].

Presidente Prudente (22°07′33″ S, 51°23′20″ W) has been considered a city with a high-quality of life in Brazil, ranked as 138th in population with 225,668 inhabitants (top 2.3%), 83rd in per capita income with monthly earnings of USD 205.75 (1.5%), and 25th on the Human Development Index with 0.806 (top 0.45%) out of the 5570 Brazilian cities, in the last 2022 nationwide census [20]. City climate has been classified as Aw (Koppen), with rainy warm summers (average 31 °C) and mild–cold dry winters (average 26.5 °C), with 1340 mm pluviometry in average per year.

The sample size was estimated, based on a previous serosurvey, for toxocariasis in blood donors in Western São Paulo [21]. A Scalex SP calculator (v. 1.0.01) was adopted for prevalence studies [22], according to the following parameters: a 13.0% seroprevalence in blood donors (considering the superior limit of the 95% confidence interval); ±4.0% error, and a 5.0% of information potential loss. The sample size was estimated to be 287, but it was adjusted to 290 participants.

The recruitment of participants, the administration of a questionnaire to obtain socio-epidemiological data, and the cognitive assessment were conducted during routine care at the Dr. João Carlos Grigoli Clinical Analysis Laboratory of the University of Western São Paulo (UNOESTE), Brazil. Firstly, individuals, or legal guardians, were invited to voluntarily participate in the study, and were informed about methodology, confidentiality, and the right of refusal anytime. Participants were asked to sign a Free and Informed Consent Term, in compliance with the Resolution No. 441/2012 of the National Health Council, Brazilian Ministry of Health. When the participant was unable to sign the document, a legal guardian signed for consent, in compliance with the Brazilian protective laws for illiterate and incapable individuals.

### 2.3. Socioepidemiological Questionnaire

A structured questionnaire (Supplementary File S1) was applied to assess the potential associated risk factors with the presence of anti-*Toxocara* spp. antibodies, based on a previous study conducted with blood donors in the same Presidente Prudente city, São Paulo state, Brazil [21].

The questionnaire consisted of socioepidemiological questions that could be associated with *Toxocara* spp. infection (Table 1). Before the interview, participants were informed about the transmission, symptoms, and prevention of toxocariasis.

### 2.4. Cognitive Assessment

The cognitive assessment was performed by the 10-point Cognitive Screener (10-point CS), with analysis of cognitive domains including temporal orientation, category fluency, and word recall, according to an established protocol [23]. Briefly, the first test comprised an evaluation of temporal orientation by asking the patient to pinpoint the year, month, and day. Secondly, the fluency test was based on asking the patient to say as many animals as possible in one minute. The word recall was assessed by providing the individual with three common words and then asking them to recall the words after a standard elapsed time.

The patient assessment results were compiled in an individual grade ranging from zero to ten points, with a higher score indicating less impairment. An individual graded with 8–10 points was considered normal, 6–7 points indicated possible cognitive impairment, and 0–5 points denoted probable cognitive impairment [23]. An adjustment for educational level was adopted by adding two points for individuals with nonformal education and one point for those who have one to three years of education. In the present study, assessment was performed by a certified geriatrician for all patients, with exception of those presenting moderate to severe dementia.

### 2.5. Blood Sampling and Testing

Participants were attended to at the Clinical Analysis Laboratory, University of Western São Paulo (UNOESTE). Blood samples (5.0 mL) were collected from all participants by a certified nurse, placed into vacuum tubes with gel separator (Vacutainer, BD Co., Curitiba, Brazil), and centrifuged at 1569× *g* for 5 min. The obtained serum was transferred to individual microtubes and stored at −20 °C until testing. Each sample was identified with a serial number to ensure anonymity and blind testing. 

The detection of anti-*Toxocara* spp. (IgG) antibodies was performed by indirect ELISA test (Enzyme-linked Immunosorbent Assay) against excretion–secretion antigens (TES), as previously established [24] and used in other research, as with blood donors [21] and pregnant women [25] of Presidente Prudente city. Briefly, the TES antigen was prepared using adult *T. canis* worms that were spontaneously released from naturally infected puppies following the protocol described elsewhere [24]. In order to reduce potential cross-reactivity with other ascarid antigens, all the serum samples were pre-adsorbed with *Ascaris suum* adult worm extracts (AWE) before the ELISA test, using a previous protocol [24,26]. Briefly, serum samples were diluted (1:200) in the pre-absorbing solution with AWE (dilution 1:200; 25 µg/mL) in PBS-Tween (Sigma, St. Louis, MO, USA) solution for 30 min at 37 °C. As previously reported, the assay exhibited 78.3% sensitivity and 92.3% specificity [27,28], and the ELISA test was performed following a published protocol [24]. Concisely, polystyrene 96-well microtiter plates (Corning, Costar, New York, NY, USA) were coated with TES and blocked with a commercial skimmed milk (Molico, Araçatuba, Brazil) in PBS-Tween solution. The pre-adsorbed serum samples were tested in duplicate and five positive controls (serially diluted), three negative controls, and seven wells were filled with threshold reactive serum in each plate. The controls used herein have been stored in the serum bank and are routinely used for serodiagnosis of toxocariasis by ELISA. Negative control sera were negative for parasites in previous studies [29,30]. The absorbance values were read at 492 nm and the global cut-off absorbance value was defined as the mean absorbance reading for the threshold reactive serum, which was set at 0.240. Serum samples presenting an absorbance value higher than the cut-off were considered positive.

The IgG avidity index (AvI) was performed by a dissociation method, using a 6 M urea solution (Merck, Darmstadt, Germany) as the denaturant agent, as previously established [31]. Expressed as percentage, AvI was calculated as the mean optical density (OD) of urea-treated/urea-untreated × 100. Values of AvI up to 50 were considered of low avidity, indicating acute infection, while AvI values exceeding 50 were considered as indicative of high avidity, suggesting chronic toxocariasis, according to the values established elsewhere for toxocariasis [32]. As previously shown, adequate frozen sample storage does not interfere with the serological results of the ELISA test. For instance, sera stored at a national repository (NHANES) was used in a retrospective toxocariasis serosurvey in the United States of America [33]. In addition, samples herein were aliquoted, kept frozen until processing, and thawed only once.

### 2.6. Statistical Analysis

The data obtained from questionnaires and serological results were gathered and tabulated in an Excel spreadsheet, and were randomly numbered 1 to 290 for each participant to ensure undisclosed identity and blind testing.

The associated risk factors with the presence of anti-*Toxocara* spp. antibodies were assessed by univariate analysis (Fisher’s exact test or Chi-square), based on the possible characteristics associated with toxocariasis (Table 1), as previously adopted [21,25]. The logistic regression coefficients were calculated using the maximum likelihood estimation. This optimization was iteratively performed through the Fisher Scoring (Newton–Raphson) method, updating the coefficient values until convergence was reached with the statistical significance of each coefficient, which were assessed using the Wald test. The Hosmer–Lemeshow test was used to assess the goodness of fit in the logistic model. To improve the final model, the predictor variables were tested for collinearity and the presence of influential values [34]. Variance inflation factors (VIF) were calculated to assess multicollinearity among the independent variables included in the logistic regression model, with VIF close to 1 indicating no evidence of problematic multicollinearity. From the regression coefficients for each predictor variable, odds ratio values were estimated per point and with a 95% confidence interval. The best-fitting model was considered the one that included significantly associated variables (*p*-value < 0.05) and minimized the Akaike Information Criterion (AIC) value.

The cognitive test results were coded as scores (0 = normal, 1 = possible impairment, and 2 = probable impairment) and analyzed using a permutation-based Kruskal–Wallis test implemented in the coin R package (v. 1.4-3) [35] to improve the analysis sensitivity and to enhance the *p*-values reliability.

*p*-values that were less than 5% (*p* < 0.05) were considered significant for all statistical analysis. Statistical analysis was conducted using the package “blloor” [36] by the R software v. 4.5.1. 2025 [37].

## 3. Results

### 3.1. Socioeconomic Characteristics of the Studied Population

The studied population was represented by 134 (46.2%) male and 156 (53.8%) female individuals, aging from 50 to 97 years old (mean: 69.7). Most of the individuals (187/290; 64.5%) declared to live with two to three monthly wages (minimum wage in Brazil at the time was USD 273.33), followed by 79 (27.2%) individuals with only one wage, and 23 (7.9%) with more than three minimum wages. Regarding the educational level, most (158/290; 54.5%) had studied for one to eight years, while 80 (27.6%) were illiterate individuals.

### 3.2. Seropositivity for Anti-Toxocara IgG Antibodies

The ELISA indirect test for anti-*Toxocara* spp. antibodies revealed a total of 89 (30.7%; 95% CI: 25.7–36.2%) seropositive individuals.

AvI was tested for in 88/89 seropositive samples, resulting in a range from 45.0 to 100% (mean: 82.6 ± 9.12). Only one-person (a 62-year-old woman) was considered to have an acute infection (AvI = 45.0%), while AvI suggested a chronic infection by *Toxocara* spp. for the other seropositive 87/88 (98.9%) participants. One sample was not sufficient for AvI testing.

**Figure 2:**
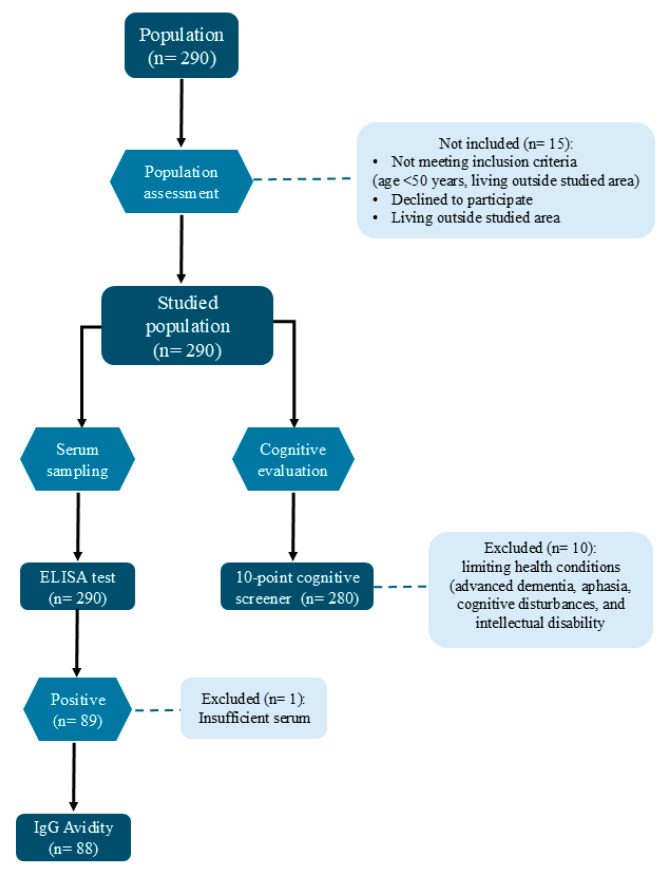
Flow-chart representing the inclusion/exclusion criteria for the population selection enrollment, serological test and avidity index, and cognitive test in individuals aging 50 years or older attended by the Public Health Service in southeastern Brazil.

A flow-chart with the inclusion/exclusion criteria, adopted for the population selection enrollment, serological test and avidity index, and cognitive test, was constructed and presented (Figure 2)

### 3.3. Risk/Protective Factors for Toxocara spp. Antibodies

Results of uni- and multivariate analysis were gathered and presented (Table 2). Only minimal and negligible missing data (*n* = 1/290, 0.34%) were observed for the variables “years of study” and “monthly income”.

A description of the Final Logistic Regression model was obtained and presented (Table 3).

The multivariate analysis revealed that individuals raising both dogs and cats were 3-fold more likely to be seropositive (*p* = 0.002) than those who declared to not have any pets, while a higher educational level was revealed as a protective factor (*p* = 0.001). 

No influence was observed between seropositivity and the other variables, including gender, monthly income, consumption of drinkable water and of raw/undercooked meat, having a dirty floor at home, contact with soil, washing vegetables and hands before meals, onychophagy, pica, and occupational exposure to soil.

No association was found between seropositivity and age considering either the interquartile distribution (*p* = 0.837; Table 2) or comparing individuals aged 50 to 65 and those older than 65 (*p* = 0.975).

No evidence of problematic multicollinearity was detected among the predictors, denoted by the very low variance inflation factors (VIF) of 1.040, 1.018, and 1.032 for the logistic regression model, respectively, for “dog and cat”, “drink water”, and “years of study”. The Hosmer–Lemeshow test yielded a chi-squared value of 6.65 with 8 degrees of freedom (*p* = 0.5751), indicating good model fit and that the null hypothesis was not rejected.

The receiver operating characteristic (ROC) curve, performed on the full sample to assess the accuracy of the multivariate logistic regression model for predicting seropositivity for anti-*Toxocara* spp. antibodies in the studied population, showed an area under the curve (AUC) of 0.704 (95% CI: 0.641–0.766), which was considered acceptable [38].

### 3.4. Seropositivity vs. Cognitive Disorder

Out of the 290 individuals tested for serology and assessed for risk factors associated with *Toxocara* spp., 280/290 (96.6%) were included in the neurocognitive screening. The remaining 10 individuals were excluded due to limiting health conditions such as advanced dementia, aphasia, cognitive disturbances, and intellectual disability. No significant differences were observed in the socioeconomic characteristics or seropositivity between excluded and included participants (Supplementary Table S1).

Most participants (111/280; 39.6%) presented possible cognitive impairment (6–7 points), followed by (87/280; 31.1%) probable cognitive impairment (0–5 points) and (82/280; 29.3%) having no detectable disorder (8–10 points) or normal individuals. No association was verified comparing the seropositivity in normal individuals (24/82; 29.3) with MCI (29/111; 26.1%; 95% IC: 0.62–2.21; *p* = 0.749) and MND (30/87; 34.5%; 95% IC: 0.41–1.50; *p* = 0.574). In addition, no statistical differences were found between cognitive scores and overall seropositivity tested by the Kruskal–Wallis permutation test (chi-squared = 0.412, *p*-value = 0.521) or among those older than 65 years (chi-squared = 1.097, *p*-value = 0.302), as presented (Table 4).

Among all of the individuals, twelve (12/290; 4.1%) had a previous diagnosis of dementia, with six seropositive and six seronegative individuals identified with the ELISA test. Neuropsychiatric disorder was verified in one third (96/290; 33.1%) of the studied population. No association was found between seropositivity and dementia (*p* = 0.198) or neuropsychiatric disorder (*p* = 0.440).

## 4. Discussion

The present study was the first serosurvey on toxocariasis in an elderly population in Brazil and addressed associated risk factors in people of 50 years of age and older. Despite the seropositivity herein (30.7%) was higher than the 20.0% worldwide estimative for adult population, the results were close to the 32% and 31% prevalence serosurveys in children and children/adults in Latin America and the Caribbean, respectively, according to a recent meta-analysis [39]. In Brazil, *Toxocara* seroprevalence has ranged from 18/280 (6.4%) pregnant women from an obstetric center in the southern region [40] to 246/258 (95.3%) indigenous individuals from a tri-border community of Brazil, Paraguay, and Argentina [41]. In this study, despite the absence of risk factors, the expressive amount of infective eggs found in soil has highlighted the role of environmental exposure in sustaining transmission in this community [41]. This result also highlighted that non-dewormed dogs and cats share environments with indigenous individuals and health care professionals [41,42].

Although high exposure to contaminated soil by *Toxocara* spp. infective eggs has reportedly predisposed children to infection, justifying the high seropositivity in younger people [4], higher seropositivity was also observed in individuals over 40 years old in Slovakia [43] and adults aged over 21 years in Poland [44]. In addition, an increase in toxocariasis in individuals older than 50 (OR: 2.4), 70 (OR: 2.3), and 80 years of age (OR: 2.6) was observed in the USA, according to the “National Health and Nutrition Examination” database [45]. Also, anti-*Toxocara* antibodies in elderly people may be a consequence of the continuing and cumulative effects of *Toxocara* exposure during their lifetimes [46]. A previous study also conducted in the United States has evaluated approximately 1350 observations from the “National Health and Nutrition Examination” during 2013 and 2014 [47]. Although *Toxocara* seropositivity and serointensity were associated with cognitive deficit in older adults, the results have not supported the association between infection and dementia [47]. Moreover, the seropositivity rate was (but not significantly) associated with 36 to 59 year-old middle-aged individuals in China [48]. Herein, no association between seropositivity and age was observed when considering the interquartile distribution or comparing individuals aged 50 to 65 and older. Thus, 65 years old has been adopted to differentiate early to late-onset dementia [49]. 

In Brazil, high seroprevalence (71.8%) was reported in a rural adult (older than 18 years) population in the southern region, with seropositivity mostly observed in persons older than 60 years, likely by larvae reactivation due to the immunocompromised status of elderly individuals [50]. In addition, immunosenescence has been reportedly associated with an increase in susceptibility to infections and perpetuating chronic neuroinflammation [51]. Such predisposed exacerbation or reactivation may result in atypical or severe presentations, such as pulmonary toxocariasis in elderly patients under chemotherapy [52]. Finally, *Toxocara* spp. larvae may physically disrupt and impair neuronal tissue and neurotransmission [53]. 

As no statistical difference was observed herein when comparing age groups, exposure was likely an age-independent factor, corroborating previous serosurveys in adult individuals in Iran [54,55]. Nonetheless, vulnerable populations, such as *quilombola* communities living in southern Brazil, have shown that individuals over 50 years of age were 7.7-fold more likely to be seropositive when compared to children, probably due to maintenance of anti-*Toxocara* spp. antibody levels over time caused by exposure to infection via soil in daily agriculture activities [56]. Thus, urban settings of attendance herein may have led to a different toxocariasis profile for the enrolled elderly population.

Despite the individuals herein mostly (168/290, 57.9%) declared having a dirty floor at home, and 68/290 (23.4%) declared participation in agriculture activities, the variable contact with soil was not associated with seropositivity. However, a previous study in the same area has shown that contact with soil increased the odds for the seropositivity for blood donors times four [21]. Thus, seroprevalence of human toxocariasis may have varied due to different factors such as soil contamination rate, level of contact and exposure, and level of social hygiene [55].

The logistic regression revealed that high educational level was a protective factor (inversely associated) for seropositivity, corroborating the other serosurveys in Brazil including people experiencing homelessness [57] and incarcerated women [58], but not corroborating surveys of rural people [50] or populations living on oceanic islands and seashore mainland cities [59]. As expected, low education has been a risk factor for toxocariasis in the United States [45]. As educational level has been a social determinant for human toxocariasis [1], the study herein has confirmed such an influence on elderly individuals.

The presence of both dogs and cats in the household was the standalone associated risk factor for seropositivity based on the logistic regression, being 3-fold higher for a positive ELISA test. Close contact with dogs and cats has been reported as an associated risk factor with *Toxocara* seropositivity [1,60] and the main transmission form in individuals presenting neurotoxocariasis [61]. Despite seropositivity and contact with both dogs (OR = 1.53) and cats (OR = 1.64) being associated with individuals under 18 years old in a meta-analysis study, no association was found for the adult population [62]. Likewise, no association between seropositivity and contact with dogs or cats was observed with blood donors (18 to 65 years old) living in the same region [21]. This result corroborated a previous study that indicated a higher prevalence of *Toxocara* seropositivity in aged people older than 60 years old with the presence of dogs in the household; contact with dogs is a major risk factor for the presence of *Toxocara* seropositivity in people [63]. As suggested in a review study, pets owned by elderly individuals may contribute to reduced loneliness and stress, mood improvement, and as a stimulus of human social interaction and sense of community [64]. Despite such well-known benefits, dogs and cats living in undeveloped and developing countries may not be adequately dewormed and vaccinated, contributing to zoonosis transmission and the spread of disease in elderly individuals by close contact, particularly with intestinal protozoa and other gastrointestinal agents [65].

Although considered less frequent, ingestion of *Toxocara* spp. eggs in contaminated dog hair should be considered as a transmission route for elderly people raising pets [66]. Prophylactic pet anthelmintic treatment and proper care and hygiene, especially for puppies, have been recommended to mitigate the transmission risk of toxocariasis and should be required for elderly owners [67]. In such a healthy scenario, pet ownership may benefit elderly individuals by providing companionship, a sense of purpose and meaning, a reduction in loneliness, and increasing socialization [68].

Gender, family income, and water supply were not significant associated risk factors herein. However, the male gender has been considered at higher risk for human toxocariasis than female [1,69], socioeconomic status is considered to influence the risk of infection in low-income populations, as observed in Turkey [70] and United States [69], and a history of drinking untreated water has been considered a significant risk factor for toxocariasis according to serosurveys [42,54] and metanalysis [71]. Herein, as participants were recruited from being individuals attending routine laboratory examinations, some sampling bias should be considered. Nevertheless, the male (45.8%) to female (54.2%) ratio herein was close to the general population living within the studied area.

Consumption of raw meat, a habit that has remarkably influenced the foodborne transmission of toxocariasis [71,72], was another risk factor not associated with seropositivity in the present study, corroborating other local studies with blood donors [21], pregnant women [73], and other studies in Brazil [58,59]. Only 10% of the individuals herein referred to the ingestion of raw beef meat, and participants mostly ate fish on islands and seashore areas, likely leading to a low risk of toxocariasis infection via food. Furthermore, commercial meat in the studied area has been generally handled under rigorous state or federal sanitary inspections [25].

*Toxocara* seroprevalence has been reportedly associated with neuropsychiatric disorders [70], likely by migration of *Toxocara* spp. larvae, which can persist in the nervous system for a long period, causing cognitive impairment and dementia related to inflammatory and chronic processes [74]. Although no association was observed considering seropositivity and neurocognitive disorder using the 10-point CS [23], such a test has shown an adequate performance in low-educated samples, as individuals herein mostly self-declared to live with a low monthly familiar income and low educational level. Neurotoxocariasis has predominantly affected middle-aged subjects and, less frequently, children < 18 years with a median age of 42 years, triggering a chronic neurological disorder, according to a literature review [61]. The results of the comparison across cognitive categories should be interpreted with caution. Although a more robust statistical methodology was applied, the power of the permutation-based Kruskal–Wallis test was considered low (approximately 5%), indicating that larger sample sizes would be required to better estimate the effect of seropositivity on cognition. Furthermore, the exclusion of 10 individuals presenting health limitations may have slightly affected the statistical analysis power, when comparing frequencies across the cognitive categories. Thus, despite no associations herein, future studies on toxocariasis and immunosenescence should be conducted to fully establish the neurological impact of the disease.

The present study has limitations, particularly in that IgG antibodies often persist for years at an elevated level, and ELISA may not be reliable to differentiate between an active and persistent infection [44]. A longitudinal study with patients clinically diagnosed with toxocariasis detected circulating IgG antibodies by ELISA over a 5-year period [75]. The avidity index (AvI) has been an alternative test for differentiating chronic acute *Toxocara* spp. infection, presenting sensitivity of 43.8% and specificity of 83.3% [43]. Herein, results suggested that 98.9% of the seropositive participants tested for AvI were considered to have a chronic infection, corroborating a study with 196 serum samples collected from 163 children with clinical, hematological, and serological evidence of toxocariasis in which 94.1% of seropositive samples presented high IgG avidity values [32]. Only one person herein was considered to have an acute infection (AvI = 45.0%). This result should be interpreted with caution, as infection may have been acquired at a younger age instead of over the elderly years. Furthermore, due to the low sensitivity of AvI, the method may be less reliable in discriminating between acute and chronic infections [32]. Reinfection was not measured and should also be considered as limitation. The present serosurvey was conducted in individuals who underwent blood sampling for routine exams, with no assessment of possible clinical signs or symptoms during blood collection. Furthermore, more detailed data (e.g., number and age of pets per household, deworming history, etc.) were not assessed to avoid a time-consuming questionnaire and interference on the time of cognitive evaluation. Herein, no stool examination of the population was performed to assess the co-infection by *Ascaris lumbricoides* or other helminths, which could interfere in the ELISA and produce a false increase in the seroprevalence results. However, serum samples were pre-adsorbed with *A. suum* adult worm extract to mitigate cross-reactivity with other Ascaridia, as previously established [26] and widely adopted in other toxocariasis serosurveys [25,41,42,73]. Furthermore, a study conducted in Brazil with children has shown that the majority of patients (84%) were infected by intestinal parasites, but such an infection had no influence on the IgG-ELISA test for toxocariasis, based on the same pre-adsorption protocol used herein [24]. The ROC curve was generated using the full dataset, which may have led to an optimistic AUC. This was an acceptable limitation, as cross-validation was constrained by the relatively small sample size, and the primary aim of the study was to assess the associated factors rather than to use the model for prediction. Finally, questionnaire answers may have been biased due to question misunderstanding and forgetfulness, particularly in such elderly individuals who could have neurocognitive disorders.

## 5. Conclusions

In conclusion, toxocariasis has been likely under-reported and neglected in the elderly population. Considering the high seroprevalence observed herein in a group at risk of immunosenescence, this age group should be considered and monitored. Furthermore, hygiene and the deworming of pets belonging to old persons should be encouraged for reducing the risk of toxocariasis transmission.

## Figures and Tables

**Table 1:** Gathered information to assess possible risk factors associated with toxocariasis in individuals 50 years of age and older.

Topics	Information
Socioeconomic characteristics	Gender, age, educational level, and family income
Pets	Contact with dog and/or cat
Hygiene habits	Handwashing before meals
Habits	Contact with soil, ingestion of raw/undercooked meat, and nail biting

**Table 2:** Associated risk factors for anti-*Toxocara* spp. (IgG) antibodies in individuals aging 50 years or older attended by the Public Health Service in southeastern Brazil (*n* = 290), by univariate and multivariate (logistic regression) statistical analysis.

	Positive	Negative	Statistical Analysis			
	*n* (%)	*n* (%)	Univariate		Multivariate	
	89 (30.7)	201 (69.3)	OR (95% CI)	*p*	OR (95% CI)	*p*
Age						
50–59	13 (14.6)	38 (18.9)	1.0 [Reference]	0.837		
60–69	33 (37.1)	71 (35.3)	1.35 (0.64–2.96)			
70–79	29 (32.6)	60 (29.9)	1.40 (0.66–3.12)			
>79	14 (15.7)	32 (15.9)	1.27 (0.52–3.16)			
Gender				0.493		
Female	45 (50.6)	112 (55.7)	1.0 [Reference]			
Male	44 (49.4)	89 (44.3)	1.23 (0.74–2.03)			
Years of study *				0.004		
Illiterate	35 (39.3)	45 (22.5)	1.0 [Reference]		1.0 [Reference]	
1–4	31 (34.8)	74 (37.0)	0.54 (0.29–1.00)		0.49 (0.25–0.92)	0.027
5–8	16 (18.0)	37 (18.5)	0.56 (0.26–1.16)		0.51 (0.23–1.08)	0.085
>8	6 (6.74)	34 (17.0)	0.21 (0.08–0.50)		0.19 (0.07–0.48)	0.001
Monthly income				0.306		
1 minimum wage	27 (30.7)	52 (25.9)	1.0 [Reference]			
2–3 minimum wage	57 (64.8)	130 (64.7)	0.84 (0.48–1.49)			
>3	4 (4.55)	19 (9.45)	0.42 (0.11–1.27)			
Drinkable water				0.121		
No	48 (53.9)	87 (43.3)	1.0 [Reference]		1.0 [Reference]	
Yes	41 (46.1)	114 (56.7)	0.65 (0.39–1.08)		0.67 (0.39–1.15)	0.146
Dirty floor at home				0.467		
No	39 (43.8)	99 (49.3)	1.0 [Reference]			
Yes	50 (56.2)	102 (50.7)	1.24 (0.75–2.06)			
Contact with soil				0.617		
No	35 (39.3)	87 (43.3)	1.0 [Reference]			
Yes	54 (60.7)	114 (56.7)	1.18 (0.71–1.97)			
Raising dog and/or cat				0.001		
None	30 (33.7)	83 (41.3)	1.0 [Reference]		1.0 [Reference]	
Dog	28 (31.5)	69 (34.3)	1.12 (0.61–2.07)		1.02 (0.54–1.91)	0.957
Cat	4 (4.49)	24 (11.9)	0.48 (0.13–1.37)		0.43 (0.12–1.27)	0.156
Dog and cat	27 (30.3)	25 (12.4)	2.96 (1.49–5.95)		3.09 (1.52–6.38)	0.002
Onychophagy				0.279		
No	85 (95.5)	183 (91.0)	1.0 [Reference]			
Yes	4 (4.49)	18 (8.96)	0.49 (0.14–1.38)			
Pica				0.768		
No	84 (94.4)	192 (95.5)	1.0 [Reference]			
Yes	5 (5.62)	9 (4.48)	1.29 (0.38–3.91)			
Raw/undercooked meat consumption				1.0		
No	77 (86.5)	173 (86.1)	1.0 [Reference]			
Yes	12 (13.5)	28 (13.9)	0.97 (0.45–1.98)			
Washing vegetables				0.342		
Water	65 (73.0)	130 (64.7)	1.0 [Reference]			
Vinegar	21 (23.6)	58 (28.9)	0.73 (0.40–1.29)			
Sodium hypochlorite	3 (3.37)	13 (6.47)	0.48 (0.10–1.58)			
Washing hands before meals				0.704		
No	3 (3.37)	5 (2.49)	1.0 [Reference]			
Yes	86 (96.6)	196 (97.5)	0.72 (0.17–3.79)			
Laboral exposure to soil				0.624		
No	66 (74.2)	156 (77.6)	1.0 [Reference]			
Yes	23 (25.8)	45 (22.4)	1.21 (0.67–2.15)			

* Years of study (calculated based on 289 individuals due to one missing data point). 1.0 [Reference]: reference for the variable category; OR (95% CI): odds ratio (95% Confidence Interval). OR (95% CI) and *p* values in Multivariate Analysis were only calculated for variables in which *p* value in univariate was lower than 0.2.

**Table 3:** Coefficients of variables included in the logistic regression model.

Coefficients	Estimate	Std. Error	z Value	Pr (>|z|) *
(Intercept)	−0.159	0.331	−0.479	0.632
Raising dog and/or cat				
None	Reference			
Dog	0.017	0.321	0.054	0.957
Cat	−0.848	0.599	−1.417	0.156
Dog and Cat	1.130	0.364	3.100	0.002
Drinkable water				
No	Reference			
Yes	−0.397	0.273	−1.455	0.146
Years of study				
Illiterate	Reference			
1–4	−0.719	0.325	−2.216	0.027
5–8	−0.675	0.391	−1.725	0.084
>8	−1.636	0.481	−3.403	0.001

* Statistical significance of z-value based on the standard normal distribution.

**Table 4:** Evaluation of association between seropositivity for anti-*Toxocara* spp. antibodies (IgG) and cognitive impairment in individuals aged 50 years or older attended by the Public Health Service in southeastern Brazil (*n* = 280).

	Seropositive	Seronegative		
	*n* = 83	*n* = 197	OR (95% CI)	*p*. Overall
Cognitive impairment				0.443
Normal	24 (28.9)	58 (29.4)	Ref.	
Possible	29 (34.9)	82 (41.6)	0.85 (0.45–1.63)	
Probable	30 (36.1)	57 (28.9)	1.27 (0.66–2.45)	

## Data Availability

All relevant data are within the manuscript.

## Supplementary Materials

The following supporting information can be downloaded at: https://www.mdpi.com/article/10.3390/pathogens14111095/s1, File S1: Structured questionnaire to assess the potential risk factors associated with anti-*Toxocara* spp. antibodies in elderly individuals; Table S1: Socioeconomic characteristics and seropositivity for *Toxocara* spp. of individuals aged 50 years or older attending the Public Health Service in southeastern Brazil, included and excluded from neurocognitive screening for health reasons (*n* = 290).
