# Supplementary material for: Toxocariasis as an Elderly Zoonosis: Seroprevalence, Neurocognitive Assessment, and Associated Risk Factors in Persons 50 Years and Older
Source: Pathogens. 2025 Oct 28;14(11):1095. doi: 10.3390/pathogens14111095 (PMC12654985; doi:10.3390/pathogens14111095)
Supplement: Supplementary file 1 [file pathogens-14-01095-s001.zip › pathogens-3812023-Supplementary File S1.pdf]

Supplementary File S1. Structured questionnaire to assess the potential risk factors associated with anti-*Toxocara* spp. antibodies in elderly individuals.

Participant name: ____

Laboratory identification: ____

Birty date: ____/____/____ Age:______ Sex: ( ) F ( ) M

Address: ____

____

Celphone: ( ) __________________________________

1 –Educational level?

( ) illiterate ( ) elementary ( ) high school ( ) college

Years of study: ________

2 – employed? ( ) Yes ( ) No- Type of job:___________________

3 – Retired? ( ) Yes ( ) No – Previous job:_________________________

4 – Monthly family income (minimum age)?

( ) 1 ( ) 2 to 3 ( ) 4 or more ( ) did not answer

5 – Is there piped water at home? ( ) Yes ( ) No

6 – Drink filtered water? ( ) Yes ( ) No

7 –Is there bathroom at home? ( ) Yes ( ) No

8 – Is the bathroom connected to the sewage system? ( ) Yes ( ) No

9 – Is there backyard? ( ) Yes ( ) No

Contact with soil? ( ) Yes ( ) No

10 –Dog owner? ( ) Yes ( ) No - How long? ___________________

If not, do you contact with dog of other person? ( ) Yes ( ) No

11 – Cat owner? ( ) Yes ( ) No - How long? ___________________

If not, do you contact with dog of other persons? ( ) Yes ( ) No

12 –Do you have the onychophagy habit? ( ) Yes ( ) No

13 – Do you have a habit of putting non-edible objects in your mouth? ( ) Yes ( ) No

14 – Do you usually eat meat or innards (e.g. liver) rare/raw? ( ) Yes ( ) No

15 – How are fruits and vegetables cleaned before consumption?

( ) Do not wash ( ) Just water ( ) Hypochlorite ( ) Vinager ( ) Other: _______

16 –Do you wash the hands before meal? ( ) Yes ( ) No

17 – Do you have any neuropsychiatric illness? ( ) Yes ( ) No

Which? _______________________________________ ( ) Don't know how to answer

Presidente Prudente, _____ ____________________ 202__.

____

Name and signature of participant
