# Supplementary material for: Toxocariasis as an Elderly Zoonosis: Seroprevalence, Neurocognitive Assessment, and Associated Risk Factors in Persons 50 Years and Older
Source: Pathogens. 2025 Oct 28;14(11):1095. doi: 10.3390/pathogens14111095 (PMC12654985; doi:10.3390/pathogens14111095)
Supplement: Supplementary file 1 [file pathogens-14-01095-s001.zip › pathogens-3812023-Supplementary Table S1.pdf]

Supplementary Material

Supplementary Table S1 – Socioeconomic characteristics and seropositivity for *Toxocara* spp. of individuals aged 50 years or older attending the Public Health Service in southeastern Brazil, included and excluded from neurocognitive screening for health reasons (n = 290).

	Excluded	Included	
	n (%)	n (%)	
	10 (3.4)	280 (96.6)	p*
**Anti-*Toxocara* spp (IgG)**			0.076
Negative	4 (40.0)	197 (70.4)	
Positive	6 (60.0)	83 (29.6)	
**Age**			0.468
50 - 59	1 (10.0)	50 (17.9)	
60 - 69	2 (20.0)	102 (36.4)	
70 - 79	4 (40.0)	85 (30.4)	
> 79	3 (30.0)	43 (15.4)	
**Gender**			0.752
Female	6 (60.0)	151 (53.9)	
Male	4 (40.0)	129 (46.1)	
**Years of study**			0.081
Illiterate	6 (60.0)	74 (26.5)	
1 - 4	3 (30.0)	102 (36.6)	
5 - 8	0 (0.00)	53 (19.0)	
>8	1 (10.0)	50 (17.9)	
**Monthly income**			0.156
1 minimum wage	5 (50.0)	74 (26.5)	
2 - 3 minimum wage	5 (50.0)	182 (65.2)	
>3	0 (0.00)	23 (8.24)	

* p-value for Fisher´s Exact Test
